# Epicardial Adipose Tissue and Left Ventricular Systolic Function in Rheumatoid Arthritis Assessed by Two-Dimensional Speckle Tracking Echocardiography

**DOI:** 10.7759/cureus.60495

**Published:** 2024-05-17

**Authors:** Tinglin Wang, Gehong Peng, Hongyu Liang, Bingxu Zhao

**Affiliations:** 1 Department of Echocardiography, The Affiliated Hospital of Zunyi Medical University, Zunyi, CHN

**Keywords:** left ventricle, systolic function, speckle tracking echocardiography, epicardial adipose tissue, rheumatoid arthritis

## Abstract

Introduction

Epicardial adipose tissue (EAT) is an emerging cardiovascular biomarker. Subclinical left ventricular (LV) systolic dysfunction is common in rheumatoid arthritis (RA). The aim of this study was to assess LV systolic function using two-dimensional speckle tracking echocardiography (2D-STE) and investigate its association with EAT in RA patients without clinical cardiovascular disease (CVD).

Methods

60 RA patients without manifestations of CVD and 60 age- and gender-matched healthy controls have been recruited for the study. We assessed LV systolic function and EAT in all subjects using conventional echocardiography and 2D-STE. EAT was measured as the relative echo-free region between the free wall of the right ventricle and the visceral layer of the pericardium at end-systole.

Results

Global longitudinal strain (GLS) was decreased and EAT was increased in the RA group compared to the control group. GLS was reduced as EAT increased in RA patients (r=-0.273, P=0.035). After adjusting for confounders, multivariate linear regression analysis revealed a weakened correlation between EAT and GLS.Age and disease activity scores28 were independent factors influencing GLS in RA.

Conclusion

RA patients have significantly thickened EAT compared with controls. 2D-STE can detect early LV myocardial systolic dysfunction in RA, as shown by lower GLS. Accumulation of EAT is associated with lower GLS, but older age and higher disease activity may play a greater role in LV myocardial systolic dysfunction in RA.

## Introduction

Rheumatoid arthritis (RA) is a chronic autoimmune disease that causes damage to patients' tissues and organs, affecting 0.5-1 percent of the global population, and the prevalence and incidence of RA are still increasing [1]. Apart from joint symptoms such as swelling, pain, and deformity, RA can also affect extra-articular organs such as the lungs, gastrointestinal tract, and kidneys, among others, with the heart being a crucial target organ. Cardiovascular disease (CVD) is an important cause of premature death in RA, and RA patients have a higher risk of asymptomatic ischemic heart disease, heart failure, and sudden cardiac death than the general population [2]. Cardiovascular risk prediction algorithms derived from traditional cardiovascular risk factors, such as hypertension and hyperglycemia, have demonstrated limited predictive value for CVD risk in patients with RA [3].

Epicardial adipose tissue (EAT) has received increasing attention as an emerging quantifiable marker of cardiovascular risk [4]. Abnormal deposition of EAT disrupts the anti-inflammatory and pro-inflammatory balance, exacerbates oxidative stress, and leads to atherosclerosis; the lipotoxicity of EAT can also cause myocardial damage, resulting in fibrosis of atrial and ventricular muscle and ultimately causing adverse effects such as alterations in cardiac structure and function [5]. EAT was found to be independently associated with subclinical left ventricular (LV) systolic dysfunction in a large community-based population-based study, independent of other cardiovascular risk factors [6]. Several studies have reported that RA patients have more EAT [7-9]. However, the relationship between EAT and LV systolic function in RA patients remains unclear. The two-dimensional speckle tracking technique (2D-STE) is a novel method for the early assessment of subclinical myocardial dysfunction. This study aimed to assess LV systolic function by 2D-STE in RA patients without clinical CVD and to investigate its relationship with EAT.

## Materials and methods

Study population

This study included 60 consecutive RA patients admitted to our hospital's rheumatology ward between November 2022 and December 2023 as well as 60 healthy volunteers matched by sex and age during the same period. The inclusion criteria for the RA group were between 40 and 70 years of age, and they met the 2010 American College of Rheumatology and European League Against Rheumatism classification criteria [10]. Exclusion criteria were congenital heart disease, coronary heart disease, cerebrovascular and peripheral artery disease, heart failure, cardiomyopathy, moderate to severe valvular disease, serious arrhythmia, pericardial effusion, chronic liver and kidney diseases, diabetes mellitus, hypertension, malignant tumors, severe infections, and combinations of other autoimmune diseases. Hypertension was defined as systolic blood pressure (SBP) ≥ 140 mmHg and/or diastolic blood pressure (DBP) ≥ 90 mmHg or the use of antihypertensive drugs. Diabetes mellitus was defined as fasting plasma glucose (FPG) ≥7.0 mmol/L or treatment for diabetes. Ethical approval for this study was obtained from the Ethics Committee of the Affiliated Hospital of Zunyi Medical University (approval number: KLLY-2022-115). Informed consent was obtained from the participants.

Clinical data collection

Sex, age, height, weight, waist circumference (WC), body mass index (BMI), SBP, and DBP were obtained for all subjects. WC was measured while the subject was in the standing position, from the narrowest level between the lower edge of the ribs to the anterior superior iliac spine. BMI was calculated as weight in kilograms divided by the square of height in meters. Blood samples were collected from all subjects after fasting for at least eight hours, and FPG, triglycerides (TG), total cholesterol (TC), high-density lipoprotein cholesterol (HDL-C), low-density lipoprotein cholesterol (LDL-C), C-reactive protein (CRP), and erythrocyte sedimentation rate (ESR) were recorded. Rheumatoid factor (RF) and anti-cyclic citrullinated peptide antibody (ACCP) were also obtained in RA patients. RF positivity was defined as RF ≥20 IU/mL, and ACCP positivity was defined as ACCP ≥17 U/mL. Disease activity was calculated based on disease activity score 28 joints (DAS28) using CRP in RA patients [11].

Conventional echocardiography examination

All subjects underwent transthoracic echocardiography examination connected to a limb-lead electrocardiogram in the left lateral decubitus position. A qualified sonographer, who was unaware of the subjects' data, used an S5-1 probe and a Philips EPIQ CVx ultrasound machine (Philips Medical Imaging, Andover, MA) to examine patients. LV interventricular septal thickness in end-diastolic period (IVSD), LV posterior wall thickness in end-diastolic period (LVPWD), LV end-diastolic diameter (LVEDD), and LV end-systolic diameter (LVESD), mitral annular peak systolic velocity (S′), and LV ejection fraction (LVEF) were measured according to standardized measurement methods [12].

Echocardiographic measurement of EAT thickness

**Figure 1:**
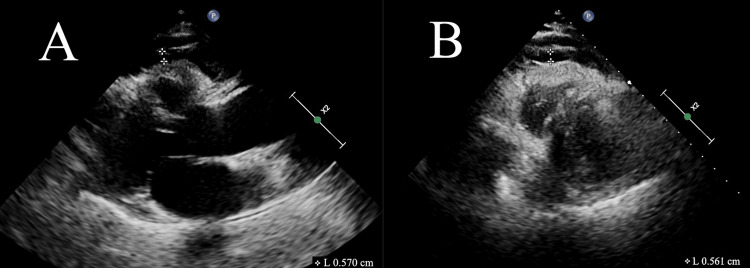
Echocardiographic measurement of EAT thickness in the parasternal long-axis view (A) and parasternal short-axis view (B) of the left ventricle at end-systole from an RA patient. Abbreviations: EAT, epicardial adipose tissue; RA, rheumatoid arthritis

Measurements were taken from both the parasternal long-axis view (Figure 1A) and the parasternal short-axis view (Figure 1B). The electrocardiogram used the T-wave endpoint as a marker of ventricular end-systole. The relatively echo-free area between the outer edge of the myocardium and the visceral layer of the pericardium was measured perpendicular to the free wall of the right ventricle at end-systole. Both views were measured three times, and the mean value was used for the analysis [13]

2D-STE examination

**Figure 2:**
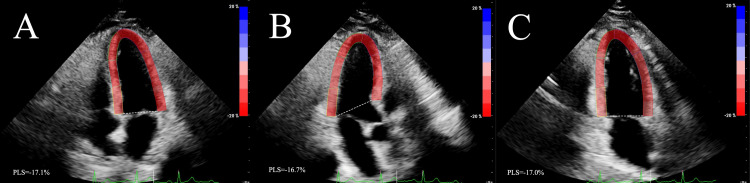
The measurement of PLS in the apical four-chamber (A), apical long-axis (B), and apical two-chamber (C) views from an RA patient. Abbreviations: PLS, peak longitudinal strain; RA, rheumatoid arthritis

Subjects breathed calmly, and dynamic images of three cardiac cycles in apical two-chamber, apical long-axis, and apical four-chamber long-axis views were acquired and stored. The global longitudinal strain (GLS) was measured using the aCMQ technique (QLAB, version 13.0; Figure 53, Baltimore, MD). The dynamic images of the three sections were matched to the corresponding views in the software, which automatically recognized the cardiac cycle, mapped the region of interest, and adjusted the region of interest if necessary. If more than two myocardial segments were not tracked satisfactorily in a single section, the subject was excluded. The software automatically outputs PLS (peak longitudinal strains) of the apical four-chamber (Figure 2A), apical long-axis (Figure 2B), and apical two-chamber (Figure 2C) views. GLS is the average of the PLS of the three views over two different cardiac cycles [14]. Because the LV myocardium shortens along its longitudinal axis during a period of contraction, and GLS describes the deformation of the myocardium in the longitudinal direction, the value is negative. To avoid confusing descriptions, it is expressed as an absolute value in this study, which means that lower absolute values of GLS represent weaker ventricular systolic function.

Repeatability analysis

Two physicians repeatedly measured GLS and EAT in 15 randomly selected subjects. One of the observers took two measurements with a one-week interval between measurements, while the second observer was unaware of the first observer's results to analyze intraobserver and interobserver variability.

Statistical analysis

Statistical Product and Service Solutions (SPSS, version 29.0; IBM SPSS Statistics for Windows, Armonk, NY) software was used for analysis. Normal distribution was expressed as mean ± standard deviation, non-normally distributed data were expressed as median (interquartile range), and categorical variables were expressed as frequencies and percentages. Comparisons between groups were made using the student t-test, Wilcoxon rank sum tests, and chi-square tests, where appropriate. Pearson and Spearman correlations were used to assess the correlation between GLS and other parameters in RA patients. Variables that were statistically significant in univariate analyses were used in multivariate linear regression analyses to explore independent associations of GLS in RA. The intraclass correlation coefficient (ICC) was used to assess the intraobserver and interobserver variability of GLS and EAT, and ICC ≥0.75 means good reliability. A two-tailed P value <0.05 was statistically significant.

## Results

Approximately 258 RA inpatients were screened, of whom 64 were initially recruited, but four were later excluded from the final analysis because unclosed patent foramen ovale was found on echocardiography in one patient, lung cancer was found during the study in one patient, and two other patients had poor image quality.

The clinical and laboratory characteristics of all subjects are shown in Table 1. There were no statistical differences between the RA group and the control group in terms of Gender, Age, BMI, SBP, DBP, FPG, TG, TC, HDL-C, and LDL-C (all P>0.05). WC, ESR, and CRP were higher in the RA group compared to the control group (all P<0.05).

**Table 1:** Comparison of clinical and laboratory characteristics in two groups (N=60) Data were expressed as Mean ± SD unless stated otherwise. ^a^Data were expressed as a number with percentage inside the bracket. ^b^Data were expressed as median (IQR). *P value<0.05; **P value <0.001 Abbreviations: RA, rheumatoid arthritis; BMI, body mass index; WC, waist circumference; SBP, systolic blood pressure; DBP, diastolic blood pressure; FPG, fasting plasma glucose; TG, triglyceride; TC, total cholesterol; HDL-C, high-density lipoprotein cholesterol; LDL-C, low-density lipoprotein cholesterol; ESR, erythrocyte sedimentation rate; CRP, C-reactive protein; DAS28, disease activity score 28 joints; RF, rheumatoid factor; ACCP, anti-cyclic citrullinated peptide antibody; SD, standard deviation; IQR, interquartile range

	Control group	RA group	P value
Woman (n/%)	48 (80.0)^a^	47 (78.3)^a^	0.822
Age (years)	54.0±7.5	53.9±5.8	0.924
BMI (kg/m^2^)	22.9±2.1	23.7±4.0	0.143
WC (cm)	80.8±4.8	85.7±9.8	0.001^*^
SBP (mm/Hg)	121.4±8.2	123.6±8.6	0.165
DBP (mm/Hg)	74.2±6.6	75.2±6.6	0.439
FPG (mmol/L)	4.9±0.4	4.9±0.5	0.950
TG (mmol/L)	1.3±0.4	1.2 (0.86)^b^	0.702
TC (mmol/L)	4.7±0.6	4.5±0.9	0.171
HDL-C (mmol/L)	1.2±0.2	1.1±0.3	0.067
LDL-C (mmol/L)	2.8±0.5	2.7±0.7	0.543
ESR (mm/h)	8.8±3.6	80.5 (53.7)^b^	<0.001^**^
CRP (mg/L)	1.4 (2.0)^b^	34.8 (76.5)^b^	<0.001^**^
DAS28	-	4.3±0.9	-
RF positive	-	53 (88.3)^a^	-
ACCP positive	-	49 (81.7)^a^	-

The echocardiographic parameters of all subjects are shown in Table 2. EAT thickness was higher and GLS was lower in the RA group than in the control group (all P<0.05). There were no differences in IVSD, LVEDD, LVPWD, LVESD, S′, or LVEF between the RA group and the control group (all P>0.05).

**Table 2:** Comparison of echocardiographic parameters in two groups (N=60) Data were expressed as mean ± SD. **P value <0.001 Abbreviations: RA, rheumatoid arthritis; EAT, epicardial adipose tissue; IVSD, interventricular septal thickness in end-diastolic period; LVEDD, LV end-diastolic diameter; LVPWD, LV posterior wall thickness in end-diastolic period; LVESD, LV end-systolic diameter; S′, mitral annular peak systolic velocity; LVEF, LV ejection fraction; GLS, global longitudinal strain; SD, standard deviation

	Control group	RA group	P value
EAT (mm)	4.21±0.51	4.97±0.93	<0.001^**^
IVSD (mm)	9.28±0.38	9.32±0.44	0.563
LVEDD (mm)	44.54±2.32	45.14±2.67	0.187
LVPWD (mm)	9.04±0.41	9.08±0.48	0.624
LVESD (mm)	28.31±1.97	28.95±2.54	0.128
S′ (cm/s)	10.85±1.45	10.47±1.65	0.187
LVEF (%)	64.30±2.32	63.62±2.58	0.130
GLS (%)	20.16±1.04	18.92±1.72	<0.001^**^

Table 3 represents the correlation analysis between GLS and other variables in the RA group. In RA patients, bivariate correlation analysis showed that EAT, age, SBP, ESR, and DAS28 were negatively associated with GLS (all P<0.05).

**Table 3:** Correlation of GLS with different factors in RA patients *P value<0.05 Abbreviations: GLS, global longitudinal strain; RA, rheumatoid arthritis; EAT, epicardial adipose tissue; BMI, body mass index; WC, waist circumference; SBP, systolic blood pressure; DBP, diastolic blood pressure; FPG, fasting plasma glucose; TG, triglyceride; TC, total cholesterol; HDL-C, high-density lipoprotein cholesterol; LDL-C, low-density lipoprotein cholesterol; ESR, erythrocyte sedimentation rate; DAS28, disease activity score 28 joints

r P value EAT -0.273 0.035^*^ Age -0.355 0.005^*^ BMI -0.108 0.411 WC -0.155 0.237 SBP -0.258 0.047^*^ DBP -0.212 0.104 FPG 0.034 0.797 TG 0.140 0.285 TC 0.069 0.602 HDL-C 0.021 0.875 LDL-C 0.101 0.441 ESR -0.314 0.014^*^ DAS28 -0.420 0.001^*^

Table 4 represents a multivariate linear regression analysis of GLS in the RA group. Multivariate linear regression analysis showed that EAT and GLS correlations weakened (P>0.05), whereas age and DAS28 were independently associated with GLS in RA patients (all P<0.05).

**Table 4:** Multiple linear regression analysis of GLS in RA patients *P value<0.05 Abbreviations: GLS, global longitudinal strain; RA, rheumatoid arthritis; EAT, epicardial adipose tissue; SBP, systolic blood pressure; ESR, erythrocyte sedimentation rate; DAS28, disease activity score 28 joints

	Nonstandardized coefficients		Standardized coefficients (β)	t	P value
	B	SE			
EAT	-0.358	0.205	-0.192	-1.746	0.087
Age	-0.098	0.033	-0.331	-2.964	0.005^*^
SBP	-0.034	0.024	-0.169	-1.428	0.159
ESR	-0.008	0.007	-0.141	-1.139	0.260
DAS28	-0.485	0.234	-0.265	-2.072	0.043^*^

The ICCs of GLS and EAT in intraobserver and inter-observer were 0.94, 0.92, and 0.90, 0.87, respectively, which means good reliability for both intraobserver and interobserver readings (all P<0.05).

## Discussion

In this study, we assessed EAT and LV systolic function by 2D-STE and, for the first time, explored the relationship between EAT and GLS in RA patients.

2D-STE has no angle dependence, and GLS is more sensitive than conventional echocardiographic parameters for detecting occult myocardial dysfunction and additionally provides prognostic information about adverse cardiovascular events [14,15]. Cioffi et al. have found that subclinical LV systolic dysfunction, as defined by GLS, independently predicted all causes and cardiovascular hospitalization in RA patients [15]. Although progress has been made in the prevention and treatment of CVD in RA in recent years, EULAR still recommends regular cardiovascular risk assessment in patients with RA [3]. Myocardial dysfunction is common in RA, with a reported 45% incidence of LV systolic dysfunction in RA patients without clinical evidence of CVD [16]. A recent meta-analysis showed reduced GLS in RA patients without cardiac disease and heart failure manifestations compared to controls [17].

Several studies in different RA cohorts have observed that higher disease severity is associated with lower LV systolic function [12,14,18]. One of the studies from Asia also confirmed that disease activity assessed by DAS28 was a major predictor of LV myocardial strain injury in RA, consistent with the results of our study [14]. Furthermore, myocardial protection by reducing the degree of disease activity and controlling the inflammatory state has also been observed in the treatment of RA [19]. EULAR also recommends controlling disease activity to reduce cardiovascular risk in RA [3]. However, age as a determinant of GLS in RA was not found in a study evaluating subclinical LV systolic dysfunction in very early RA patients, which may be because the study included outpatient RA with newly diagnosed and treatment-naive compared with ours [18].

The pathophysiological mechanism of cardiovascular involvement in RA has not been clarified, but it is currently believed that chronic inflammation leads to damage of vascular endothelial cells, accelerated atherosclerosis, microcirculatory dysfunction affects myocardial function, persistent inflammation causes oxidative stress, and the increase in pro-inflammatory cytokine-induced fibroblast activity can lead to myocardial collagen deposition and interstitial fibrosis, which affects LV function [2]. It has also been suggested that changes in adipocytokine secretion patterns could intensify the inflammatory response, worsen tissue damage, and potentially link RA to CVD [20].

EAT is a specific form of visceral fat around the heart and is metabolically active, secreting adipocytokines (e.g., leptin, resistin, interleukin-6 (IL-6), and tumor necrosis factor-α (TNF-α)) [4]. The pathogenic role of adipocytokines such as leptin, resistin, IL-6, and TNF-α in atherosclerotic CVD has been suggested [21]. Previously, an animal experiment has confirmed that leptin from pericardial adipose tissue in obese rats can induce fibrosis in cardiomyocytes [22]. Because of the lack of anatomical barrier between EAT and the myocardium and coronary arteries and the sharing of the same microcirculation, the biologically active substances secreted by EAT can act on coronary arteries and myocardial tissues through paracrine and vascular secretion, aggravating inflammatory reactions and affecting vascular and myocardial structure and function [5]. An animal experiment has confirmed that changes in the secretory products of EAT can inhibit the contractile function of cardiomyocytes in experimental rats [23].

Several previous studies have compared the thickness of EAT in RA patients with controls and found that RA patients have a higher EAT, consistent with our results [7-9]. However, there are no studies on the correlation between EAT and LV systolic function in RA patients without significant CVD. In the present study, we found a negative correlation between the thickness of EAT and LV systolic function in RA patients.

The link between EAT and atherosclerosis has been observed in RA, with a significant positive correlation between EAT and arterial stiffness, and EAT was an independent predictor of elevated aortic pulse wave velocity [24]. Significantly elevated levels of leptin and resistin have been observed in RA patients, especially those with high disease activity [25,26]. A study including 192 RA patients suggests that leptin is associated with the degree of atherosclerosis and plaque formation, independently of other cardiovascular risk factors [27].

Exploring the relationship between EAT and LV systolic function in RA patients is of great clinical value. The correlation between impaired LV systolic function and EAT suggests that EAT may be an imaging marker for assessing subclinical LV systolic insufficiency in RA. A case-control study found less EAT in RA patients treated with TNF-α inhibitors [7]. Additionally, TNF-α inhibitors have been shown to play a positive role in CVD in RA [19]. However, further studies are needed to confirm whether the beneficial effects of TNF-α inhibitors on cardiovascular function are mediated through EAT. Although the accumulation of EAT was associated with reduced GLS in RA patients in the present study, the correlation was attenuated after adjustment for confounders. Age and RA disease severity may play a greater role in LV myocardial dysfunction.

Our study also has several limitations. First, the present study was a single-center cross-sectional study with a small sample size, which did not allow us to infer a causal relationship between EAT and GLS, and the results could not be extrapolated. Second, the gold standard for EAT assessment is cardiac magnetic resonance, not echocardiographic measurements, but a recent study reported a good correlation between echocardiographic and magnetic resonance measurements of EAT [28]. Moreover, there are still many limitations of 2D-STE (e.g., image quality, frame rate, and chest wall conformation), which may account for the increased intra- and interobserver variability of GLS measurements [29]. Third, because of the mostly incomplete medical records of RA patients, it was not possible to accurately obtain the duration of the disease and therapeutic medications; therefore, the impact of these factors on the study results could not be considered. Finally, limited by the conditions of the laboratory, adipokines, and cytokines were not measured in RA patients.

## Conclusions

We conclude that significant differences in the thickness of EAT were observed among participants, with increased EAT thickness in the RA group. A 2D-STE can detect early LV myocardial systolic dysfunction in RA patients, as shown by lower GLS. The bivariate correlations further illuminate the negative correlation between GLS and EAT in RA patients, as evidenced by the accumulation of EAT associated with lower GLS. However, the predictive value of EAT in GLS of RA patients does not emerge as a robust independent predictor. Subclinical LV systolic dysfunction in patients with RA seems to be more influenced by age and disease activity rather than the thickness of EAT.
